# Remineralization potential of grape seed extract hydrogels on bleached enamel compared to fluoride gel: An *in vitro* study

**DOI:** 10.4317/jced.55556

**Published:** 2019-05-01

**Authors:** Shaymaa M. Nagi, Shahinaz N. Hassan, Sameh H. Abd El-Alim, Mostafa M. Elmissiry

**Affiliations:** 1 Assistant Researcher Professor in Restorative and Dental Materials Department, Oral and Dental Research division, National Research Centre, Egypt; 2 Researcher in Restorative and Dental Materials Department, Oral and Dental Research division, National Research Centre, Egypt; 3 Assistant Researcher Professor in Pharmaceutical Technology Department, Pharmaceutical Industries Research Division, National Research Centre, Giza, Egypt; 4 Researcher Professor in Phytochemistry Department, Pharmaceutical Industries Research Division, National Research centre, Giza, Egypt

## Abstract

**Background:**

Remineralizing of bleached enamel is a common procedure that aims to compensate enamel mineral lose. This study aimed to evaluate the remineralization effectiveness of experimentally prepared grape seed extract hydrogels (GSE) compared to fluoride gel on bleached enamel.

**Material and Methods:**

Thirty extracted maxillary incisor were bleached using white smile bleaching agent. Bleached specimens were divided into three groups (10/group) according to the remineralizing agents tested: [GSE 6%, GSE 10%, or fluoride gel]. After bleaching and remineralization procedure, the specimens were stored in artificial saliva at 37°C. Micro-hardness and Energy-Dispersive X-ray and ultra-morphological evaluation were tested at baseline, after bleaching and after remineralization.

**Results:**

Statistical significant decrease on mean micro-hardness values had resulted after bleaching procedure compared to baseline, followed by a significant increase in GSE (10%) and fluoride groups. GSE (6%) application showed the least statistical significant mean micro-hardness, which was statistically insignificant different compared to bleaching procedure. Elemental analysis results revealed a statistical significant decrease on Ca, and Ca/P ratios (At%) values after bleaching compared to baseline, followed by a significant increase after treatment with all tested remineralizing gels. SEM photomicrograph of sound enamel shows smooth uniform appearance with few pores. Bleached enamel showed irregular pitted disorganized enamel surface. Bleached enamel exposed to GSE and fluoride gel showed occlusion of enamel surface porosities and precipitates of different sizes.

**Conclusions:**

The specially prepared GSE hydrogels has positive effects on the remineralization process of bleached enamel making it an effective natural agent with remineralizing potential.

** Key words:**Remineralization, bleaching, grape seed extract, fluoride, enamel.

## Introduction

Increasing the awareness and concern about esthetic dentistry has resulted in the wide spread practice of teeth bleaching. Vital bleaching is considered a well-accepted and a safe procedure for treating discolored teeth resulting from both intrinsic and extrinsic staining (1).

The mechanism of bleaching agents is based upon an oxidation-reduction reaction. This reaction releases oxygen free radicals that are considered highly reactive, as they can penetrate the tooth structure through the porosities and enamel prisms breaking down the organic molecules with high molecular weight into inorganic molecules with low molecular weight creating the whitening effect (2).

Inspite of the great esthetic benefits brought to the patients following bleaching treatments, several studies reported the negative impact that might result following the use of bleaching agents on the integrity of the enamel structure concerning decreased hardness, mineral loss as well as morphological alterations of the dental hard tissues (3,4).

Several studies indicated that using remineralizing agents following bleaching procedures could repair early demineralized lesions owing to its chemical similarity with tooth minerals. It has been shown that Fluoride application could be beneficial following bleaching treatment as it forms fluorapatite on the enamel surface by covering the enamel hardness, thus promoting remineralization (5,6).

Recently, several studies has been advocated the usage of natural nutraceutical agents as Grape Seed Extract because of its beneficial antibacterial, antioxidant as well as its free radical scavenging properties (7,8).

Therefore, studying the effect of two different concentrations of prepared grape seed extract and fluoride gel on the hardness, chemistry and morphology of bleached enamel surface may be of a value. The null hypotheses investigated was that the different tested remineralizing agents applied after the bleaching procedures have no effect on enamel hardness, chemistry and morphology.

## Material and Methods

-Materials used in this study

One in office chemically activated bleaching agent (White smile power whitening 40% YF (32% mixed) Whitesmile GmbH; Germany; Lot 17250), and three remineralizing agents: Fluoride gel (Whitesmile mousse; Germany; 4.2% potassium nitrate, 1450 ppm sodium fluoride and 30% Xylitol), and two different concentrations (6 and 10%) of experimentally prepared Grape Seed Extract hydrogel were tested in this study.

-Preparation of grape seed extract 

Extraction of the grape seeds (Vitis vinifera L.)

120 gm of air dried powdered grape seeds (Vitis vinifera L.) were extracted by percolation in aqueous ethanol (70% v/v) for three consecutive times (3 x 150 ml) to ensure complete extraction. The collected filtered extracts were combined and dried under reduced pressure (45 °C) till complete dryness by means of a rotary evaporator (Heidolph VV 2000, Heidolph Instruments GmbH, Germany).

A dark brown residue was obtained and reduced to fine powder by the means of a mortar and pestle. The dry powdered extract was kept in an air tight container till further use.

-Preparation of grape seed extract hydrogel (GSE) formulations

Solutions of two concentrations of grape seed extract were prepared through dissolving 0.6, 1 g of the dried powdered extract in aqueous ethanol (20% v/v) and completing the volume to 10 ml to yield solutions of concentration 6% and 10% respectively.

The grape seed extract hydrogels were prepared through dissolving Sodium carboxymethyl cellulose (Sigma chemical co., USA), the gelling agent, in the previously prepared solutions (5% w/v) under continuous stirring at 1000 rpm using a magnetic stirrer (Wisestir MSH-30D, Daihan scientific co. Ltd., Korea). The prepared GSE hydrogels were kept at 8°C for 24 hours before further use.

-Teeth selection and specimens’ preparation

A total of thirty extracted sound maxillary incisor teeth scheduled for extraction for periodontal reason were utilized in this study. Teeth were scraped with hand scaler and washed under running tap water to remove any residual tissues and debris. A double side-cutting disc was used to cut the roots of the specimens 2mm below the cemento-enamel junction. Pulp tissue was removed using barbed broaches (4). Specimens were stored in deionized water until testing. Baseline micro-hardness testing and elemental analysis were carried out for all the thirty specimens.

-Specimens grouping

The 30 specimens received bleaching treatment using white smile chemically-activated bleaching agent. Then, bleached specimens were divided randomly into three groups of 10 specimens each according to the three different remineralizing agents used following bleaching treatment. Group A (n=10): specimens received GSE 6% ; Group B (n=10): specimens received GSE 10% (9); Group C (n=10): specimens received fluoride gel. Sample size calculation was done using R statistical package, version 2.15.2 (26-10-2012). Copyright (C) 2012 - The R Foundation for Statistical Computing. In a one-way ANOVA study, results showed that a total sample size of 10 samples will be adequate to detect a mean difference between study groups with a power of 80% and a two-sided significance level of 5%.

-Bleaching agent application

The crowns of the prepared specimens were labelled from the palatal surface, then fixed on a glass slab using double adhesive tape to facilitate the applications of the bleaching agent. White smile chemically-activated bleaching gel was applied to the labial surfaces of all prepared specimens by dispensing the material from the dual barrel syringe by attaching the mixing tip to the top of the syringe. The gel was applied in 1.5 to 2mm thickness and left for 15 minutes according to the manufacturer instructions. The gel was then removed using cotton rolls to be ready for another fresh gel mix. That procedure was repeated three times resulting in a total of 45 minutes application of the bleaching gel (10). Specimens were then removed from the glass slab, rinsed under running tap water, then stored in artificial saliva until testing. After bleaching procedure, micro-hardness testing, and elemental analysis were done.

-Application of the remineralizing agents

The three tested remineralizing agents (the experimentally prepared GSE hydrogels (6% or 10% concentration) and the Fluoride gel) were applied to the labial surfaces of the assigned bleached specimens group. The remineralizing agents were applied in 1.5 to 2mm thickness and left for 10 minutes (11) on the labial surfaces. Then specimens were rinsed under running tap water, stored in artificial saliva until testing. After reminiralizing procedure; micro-hardness testing, and elemental analysis were carried out.

-Surface Hardness measurement

Surface micro-hardness measurement was carried out using Vickers micro-hardness Tester (Shimadzu HMV-M Micro-hardness tester; Newage Testing instruments Inc., Southampton, PA, USA) under 200gm load, and 15 seconds dwell time (12). Three indentations were performed on each specimen, and the mean value of the readings was calculated and recorded. Micro-hardness measurements were obtained at baseline (before bleaching procedure), after bleaching process and after remineralization process.

-Elemental analysis and Surface morphology assessment

All specimens were subjected to elemental analysis using Environmental Energy Dispersive X-ray Analysis (EDX, Model Quanta 250, FEI Company Netherlands) to determine the level of the calcium and phosphorus ion in the enamel surface; at baseline, after bleaching process and after remineralization process. Specimens were arranged over stubs inside a chamber with their labial surfaces were facing upwards. Representative specimens were examined under scanning electron microscope (SEM) attached to EDX, to assess morphological changes at baseline, after bleaching process and after remineralization process at magnification 2000X.

-Statistical analysis

Data presented as mean and standard deviation (SD). Data explored for normality using Kolmogorov-Smirnov and Shapiro-Wilk tests. Data showed a parametric distribution, so One-Way ANOVA used to study the effect of different remineralizing agents followed by Tukey’s HSD post-hoc test for pairwise comparison. Dependent t-test used to compare with the control after bleaching. The significance level was set at P ≤ 0.05. Statistical analysis was performed with IBM® SPSS® (SPSS Inc., IBM Corporation, NY, USA) Version 25 for Windows.

## Results

Mean and standard deviation (SD) for the micro-hardness (VHN) results for tested groups are presented in  Table 1. Results revealed that a statistical significant decrease on mean micro-hardness values had resulted after bleaching procedure compared to baseline, followed by a significant increase on the mean micro-hardness in GSE (10%) and fluoride groups, where there was no statistical significant difference between them. GSE (6%) application showed the least statistical significant mean micro-hardness, which was statistically insignificant different compared to bleaching procedure.  Table 2, Table 3, Table 4 showed the mean and standard deviation (SD) for Ca, Ph and Ca/P ratios (At%) results for tested groups. Results revealed that a statistical significant decrease on Ca, and Ca/P ratios (At%) values after bleaching compared to baseline, followed by a significant increase after treatment with all tested remineralizing gels.

**Table 1:**
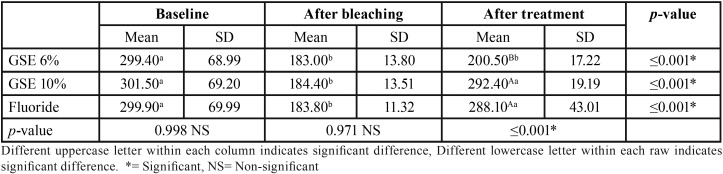
Mean, standard deviation (SD) value of Micro-hardness results (VHN) for tested groups.

**Table 2:**
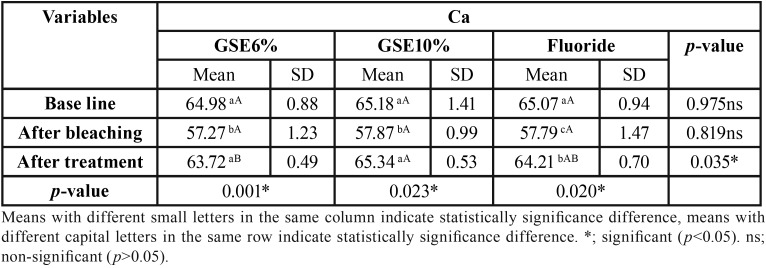
The mean, standard deviation (SD) values of Ca (At%) of different groups.

**Table 3:**
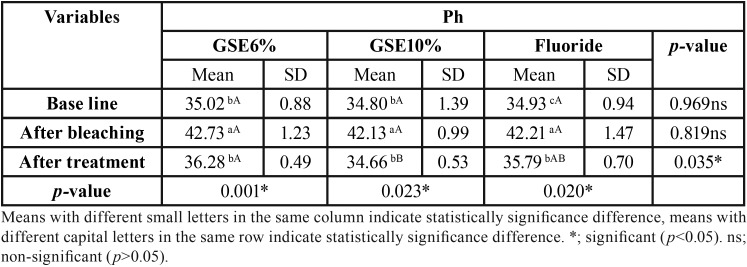
The mean, standard deviation (SD) values of Ph (At%) of different groups.

**Table 4:**
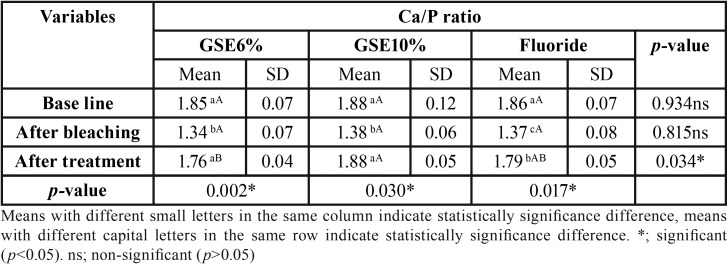
The mean, standard deviation (SD) values of Ca/P ratio (At%) of different groups.

**Figure 1:**
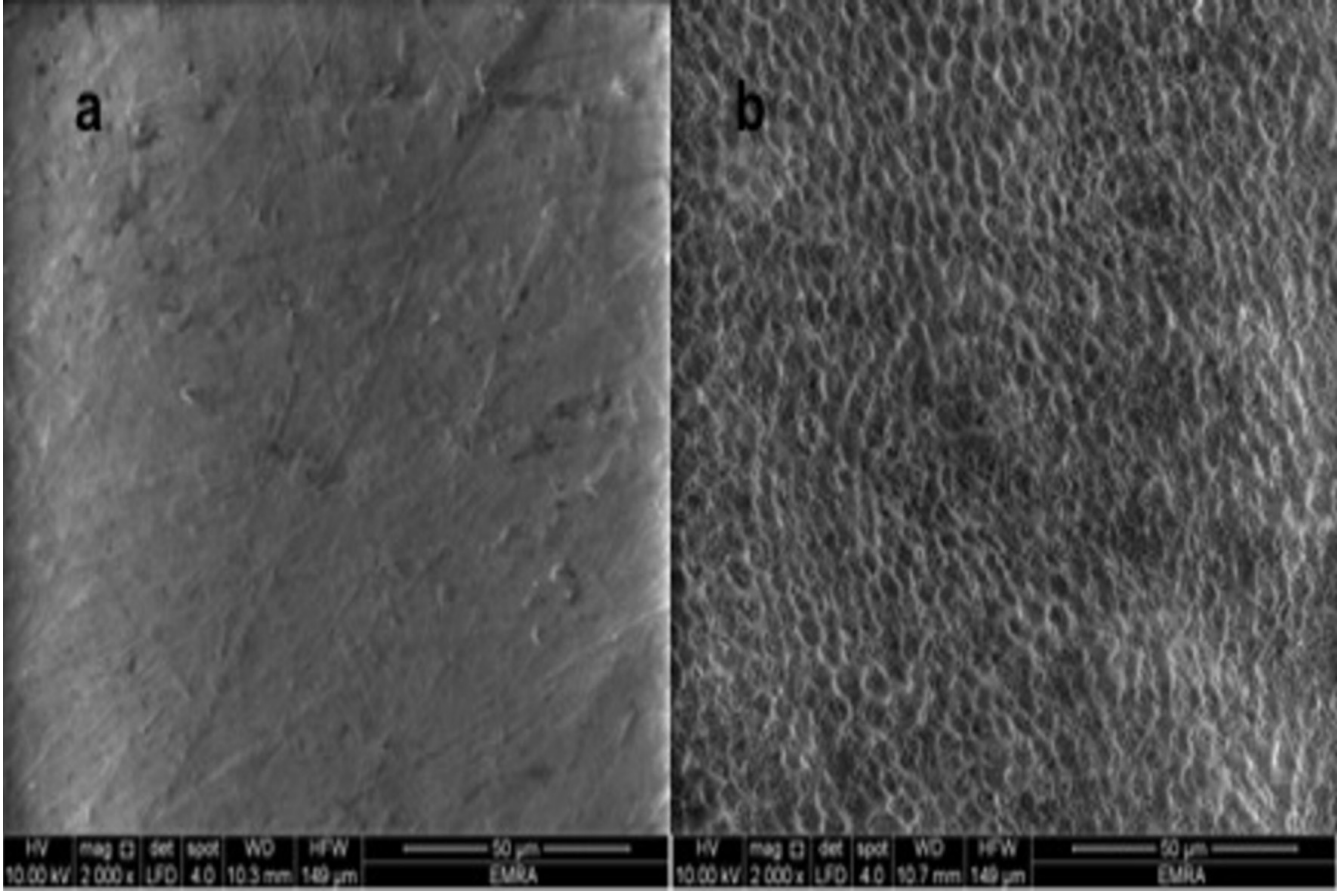
a. Untreated enamel surface (baseline), b. Bleached enamel surface.

**Figure 2:**
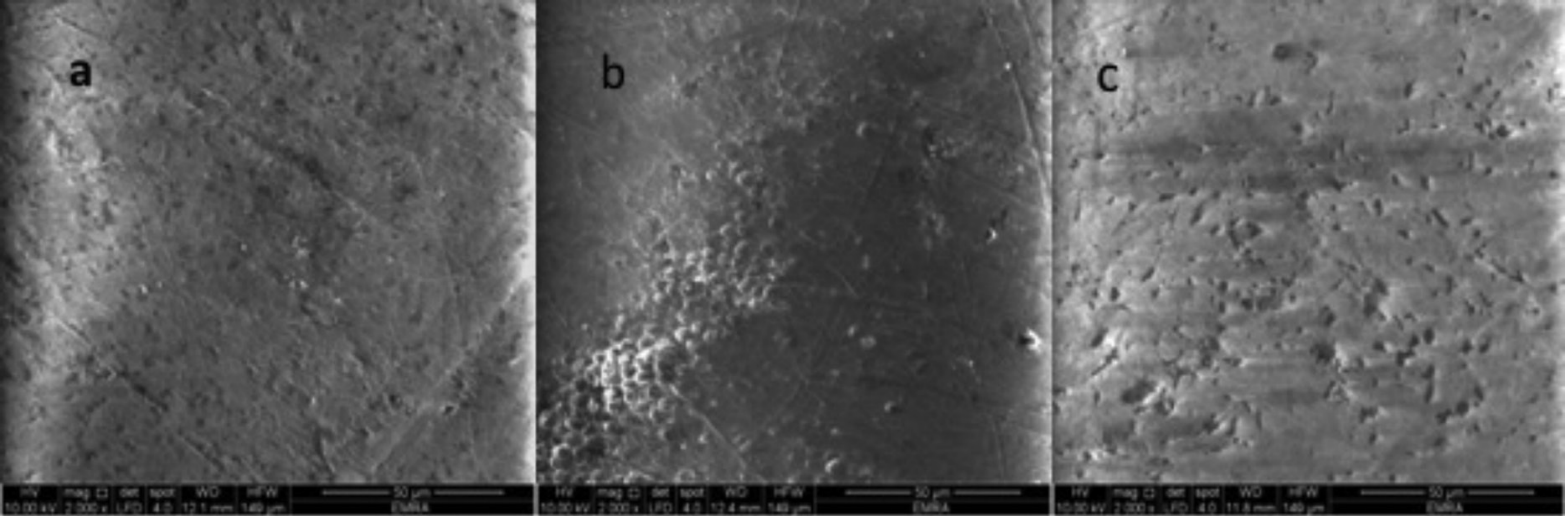
a. Enamel surface treated with GSE 6%, b. Enamel surface treated with GSE 10%, c. Enamel surface treated with Fluoride gel.

Results of the scanning electron microscope (SEM) examination (×2000 magnification): Figure 1a. shows SEM photomicrograph of sound enamel, which shows smooth uniform appearance with few pores. The aprismatic surface layer was uniform. Photomicrograph of bleached enamel before treatment shows; irregular pitted, rough and disorganized enamel surface with significant porosities was presented in Figure 1b. Figure 2a. shows bleached enamel exposed to GSE (6%), occlusion of most of enamel surface porosities and precipitates of small sizes were observed on the enamel surface. On the other hand; bleached enamel exposed to GSE (10%), shows coating depositions of some insoluble complexes on the enamel surface. The reaction products of GSE are seen as amorphous clumps. Spherical globular agglomerates were observed on the surface of the enamel, with varying sizes from place to place Figure 2b. SEM photomicrograph of bleached enamel exposed to fluoride Figure 2c, showing partially occlusion of enamel surface porosities.

## Discussion

Teeth bleaching is a process in which pigmented molecules are fragmented into smaller ones that alter the light absorption, thus reduce or exclude stains. Several studies reported that teeth bleaching modifies the chemical composition of enamel (11,13-15). Thus; remineralizing the bleached enamel surface by suppling calcium and phosphate ions from an external source is mandatory to producing net mineral gain (13).

In this study enamel micro-hardness, elemental and surface morphological analysis were tested to investigate the effect of natural nutraceuticals (presented in GSE in two different concentrations) remineralization potential compared to fluoride on bleached enamel.

Micro-hardness tester and EDX were chosen to be tested in this study as they are indicative of chemical changes. Chemical changes indicate that the mineral phase was distorted or that a significant replacement of ions may have happened (16).

In the current study, although the specimens were immersed in artificial saliva after the bleaching procedures, the micro-hardness, Ca and Ca/Ph ratio mean values were statistically significantly decreased compared to baseline. This indicates that artificial saliva alone is not sufficient to repair the bleached enamel to normal. More over irregular pitted enamel surface was revealed on bleached enamel photomicrographs (Fig. 1b). Our results were in accordance to Coceska *et al.*, in 2016 (17) and da Costa Soares *et al.*, in 2013 (18). This might be explained due to reduction of pH of the bleaching agent after its decomposition into water, oxygen, carbon dioxide, and ammonia. This low pH might dissolute the mineral content of enamel thus decreasing enamel micro-hardness, Ca and Ca/Ph ratio mean values after bleaching procedures (17,18).

Fluoride is one of the most frequently used and effective remineralizing agent after tooth bleaching. A significant increase in the mean micro-hardness, Ca, and Ca/Ph ratio after remineralization with Fluoride gel was revealed in this study. Fluoride included into the tooth surface, creating a calcium fluoride layer that increases the mineral content and hardness values of enamel surface (19).

A newer concept for remineralization is the use of natural products or nutraceuticals on the remineralization processes of dental hard tissues. The present study tested the benefits of GSE remineralizing potential in two different concentrations. Results of the present study show a significant increase in the mean micro-hardness, Ca, and Ca/Ph ratio after remineralization with GSE at both concentrations compared to bleached group. These results were supported by SEM photomicrographs of GSE specimens that showed scaffolding deposits on the enamel surface with cluster-like structures resembling remineralization process initiation specially seen when GSE was used in high concentration (10%) (figure 2b).

One of the major constituents of GSE is Gallic acid, which was supposed to facilitate mineral deposition, mainly on the surface layer (7) by combining with Ca2+ from the surrounding media and also by forming insoluble compounds with Ca (8,17,20).

GSE biological active constituents are polyphenols, mainly proanthocyanidins (PA), which are condensed tannin; it corresponds to a variation of polymers of flavan-3-ol, as catechin and epicatechin. Since GSE is a powerful source of PA, it was reported that PA increase collagen cross-links by strengthening collagen-based tissues (8).

Although it is widely known that mature enamel is considered collagen free substrate, many studies showed that type I collagen and type X collagen are present in enamel and are one of the applicant molecules found in enamel matrix (8,21,22).

Collagen is considered a proline-rich proteins with extremely high affinity for PA-based components (GSE is a powerful source of PA), forming a proline–PA complex (23,24). GSE increase the collagen cross-links by four different mechanisms: ionic, covalent, hydrogen bonding, and hydrophobic interactions (8,23,25). According to these findings, it is expected that exogenous collagen cross-links involves in the remineralization of enamel defects by GSE (8). The absorption of collagen peptides to the hydroxyapatite surfaces primary related to the terminal carboxyl groups and amine groups. Moreover, the –OH and positively charged –NH3+ groups of peptides specifically combine intensely to the surface, so their existence encourages hydroxyapatite growth (26).

The null hypotheses that the different tested remineralizing agents applied after the bleaching procedures have no effect on enamel hardness, chemistry and morphology was rejected, as there was an increase in bleached enamel micro-hardness, Ca and Ca/P ratio mean value and improvement in enamel surface morphology after treatment with the experimentally prepared GSE hydrogels and Fluoride gel.

## Conclusions

Within the limitations of the current study, it could be concluded that the specially prepared Grape seed extract hydrogels has positive effects on the remineralization process of bleached enamel making it an effective natural agent with remineralizing potential.

## References

[1] Vidhya S, Srinivasulu S, Sujatha M and Mahalaxmi S (2011). Effect of grape seed extract on the bond strength of bleached enamel. Oper Dent.

[2] Ozelin AA, Guiraldo RD, Carvalho RV, Lopes MB, Berger SB (2014). Effects of green tea application time on bond strength after enamel bleaching. Braz Dent J.

[3] Miranda TA, Moura SK, Amorim VH, Terada RS, Pascotto RC (2013). Influence of exposure time to saliva and antioxidant treatment on bond strength to enamel after tooth bleaching: An insitu study. J Appl Oral Sci.

[4] Whang HJ, Shin DH (2015). Effects of applying antioxidants on bond strength of bleached bovine dentin. Restor Dent Endod.

[5] Bizhang M, Seemann R, Duve G, Römhild G, Altenburger JM, Jahn KR (2006). Demineralization effects of 2 bleaching procedures on enamel surface with and without post-treatment fluoride application. Oper Dent.

[6] Fukuyama M, Kawamoto C, Saikaew P, Matsuda Y, Carvalho RM, Selimovic D (2017). Effect of topical fluoride application on enamel after in-office bleaching, as evaluated using a novel hardness tester and a transverse microradiography method. Eur J Oral Sci.

[7] Bedran-Russo AK, Pashley DH, Agee K, Drummond JL, Miescke KJ (2008). Changes in stiffness of demineralized dentin following application of collagen crosslinkers. J Biomed Mater Res B Appl Biomater.

[8] Mirkarimi M, Eskandarion S, Bargrizan M, Delazar A, Kharazifard MJ (2013). Remineralization of artificial caries in primary teeth by grape seed extract: An in vitro study. J Dent Res Dent Clin Dent Prospects.

[9] Subramonian R, Mathai V, Christaine Angelo JB, Ravi J (2015). Effect of three different antioxidants on the shear bond strength of composite resin to bleached enamel: An in vitro study. J Conserv Dent.

[10] Alencar MS, Bombonatti JF, Maenosono RM, Soares AF, Wang L, Mondelli RF (2016). Effect of two antioxidant agents on microtensile bond strength to bleached enamel. Braz Dent J.

[11] Sharafeddin F, Farshad F (2015). The Effect of Aloe Vera, Pomegranate Peel, Grape Seed Extract, Green Tea, and Sodium Ascorbate as Antioxidants on the Shear Bond Strength of Composite Resin to Home-bleached Enamel. J Dent (Shiraz).

[12] Patil N, Choudhari S, Kulkarni S, Joshi SR (2013). Comparative evaluation of remineralizing potential of three agents on artificially demineralized human enamel: an in vitro study. J Conserv Dent.

[13] Santini A, Pulham CR, Rajab A, Ibbetson R (2008). Effect of a 10% carbamide peroxide bleaching agent on the phosphate concentration of tooth enamel assessed by Raman spectroscopy. Dent Traumatol.

[14] Gjorgievska E, Nicholson JW (2011). Prevention of enamel demineralization after tooth bleaching by bioactive glass incorporated into toothpaste. Aust Dent J.

[15] Elfallah HM, Swain MV (2013). A review of the effect of vital teeth bleaching on the mechanical properties of tooth enamel. N Z Dent J.

[16] Soldani P, Amaral CM, Rodrigues JA (2010). Microhardness evaluation of in situ vital bleaching and thickening agents on human dental enamel. Int J Periodontics Restorative Dent.

[17] Coceska E, Gjorgievska E, Coleman NJ, Gabric D, Slipper IJ, Stevanovic M (2016). Enamel alteration following tooth bleaching and remineralization. J Microsc.

[18] da Costa Soares MU, Araújo NC, Borges BC, Sales Wda S, Sobral AP (2013). Impact of remineralizing agents on enamel microhardness recovery after in-office tooth bleaching therapies. Acta Odontol Scand.

[19] Borges AB, Yui KC, D'Avila TC, Takahashi CL, Torres CR, Borges AL (2010). Influence of remineralizing gels on bleached enamel microhardness in different time intervals. Oper Dent.

[20] Xie Q, Bedran-Russo AK, Wu CD (2008). In vitro remineralization effects of grape seed extract on artifcial root caries. J Dent.

[21] Açil Y, Mobasseri AE, Warnke PH, Terheyden H, Wiltfang J, Springer I (2005). Detection of mature collagen in human dental enamel. Calcif Tissue Int.

[22] Felszeghy S, Holló K, Módis L, Lammi MJ (2000). Type X collagen in human enamel development: a possible role in mineralization. Acta Odontol Scand.

[23] Al-Ammar A, Drummond JL, Bedran-Russo AK (2009). The use of collagen cross-linking agents to enhance dentin bond strength. J Biomed Mater Res B Appl Biomater.

[24] Han B, Jaurequi J, Tang BW, Nimni ME (2003). Proanthocyanidin: A natural crosslinking reagent for stabilizing collagen matrices. J Biomed Mater Res.

[25] Hagerman AE, Butler LG (1981). The specificity of proanthocyanidin- protein interactions. J Biol Chem.

[26] Almora-Barrios N, de Leeuw NH (2010). A density functional theory study of the interaction of collagen peptides with hydroxyapatite surfaces. Langmuir.

